# SARS‐CoV‐2 infection activates dendritic cells via cytosolic receptors rather than extracellular TLRs

**DOI:** 10.1002/eji.202149656

**Published:** 2022-02-16

**Authors:** Lieve E.H. van der Donk, Julia Eder, John L. van Hamme, Philip J. M. Brouwer, Mitch Brinkkemper, Ad C. van Nuenen, Marit J. van Gils, Rogier W. Sanders, Neeltje A. Kootstra, Marta Bermejo‐Jambrina, Teunis B. H. Geijtenbeek

**Affiliations:** ^1^ Department of Experimental Immunology Amsterdam institute for Infection and Immunity Amsterdam University Medical Centers University of Amsterdam Amsterdam The Netherlands; ^2^ Department of Medical Microbiology Amsterdam institute for Infection and Immunity Amsterdam University Medical Centers University of Amsterdam Amsterdam The Netherlands; ^3^ Department of Microbiology and Immunology Weill Medical College of Cornell University New York NY USA

**Keywords:** Dendritic cells, Innate immune response, Intracellular viral sensors, SARS‐CoV‐2, Toll‐like receptor 4

## Abstract

Severe acute respiratory syndrome coronavirus 2 (SARS‐CoV‐2) causes coronavirus disease 2019 (COVID‐19), an infectious disease characterized by strong induction of inflammatory cytokines, progressive lung inflammation, and potentially multiorgan dysfunction. It remains unclear how SARS‐CoV‐2 infection leads to immune activation. The Spike (S) protein of SARS‐CoV‐2 has been suggested to trigger TLR4 and thereby activate immunity. Here, we have investigated the role of TLR4 in SARS‐CoV‐2 infection and immunity. Neither exposure of isolated S protein, SARS‐CoV‐2 pseudovirus nor primary SARS‐CoV‐2 isolate induced TLR4 activation in a TLR4‐expressing cell line. Human monocyte‐derived DCs express TLR4 but not angiotensin converting enzyme 2 (ACE2), and DCs were not infected by SARS‐CoV‐2. Notably, neither S protein nor SARS‐CoV‐2 induced DC maturation or cytokines, indicating that both S protein and SARS‐CoV‐2 virus particles do not trigger extracellular TLRs including TLR4. Ectopic expression of ACE2 in DCs led to efficient infection by SARS‐CoV‐2 and, strikingly, efficient type I IFN and cytokine responses. These data strongly suggest that not extracellular TLRs but intracellular viral sensors are key players in sensing SARS‐CoV‐2. These data imply that SARS‐CoV‐2 escapes direct sensing by TLRs, which might underlie the lack of efficient immunity to SARS‐CoV‐2 early during infection.

## Graphical abstract

Neither TLR4‐expressing dendritic cells nor cell lines become activated by SARS‐CoV‐2. Notably, ectopic expression of ACE2 on dendritic cells leads to infection by SARS‐CoV‐2 and immune activation. Thus, SARS‐CoV‐2 triggers intracellular but not extracellular pattern recognition receptors, suggesting that immune responses are initiated upon direct infection or due to bystander inflammatory processes.

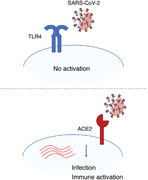

## Introduction

Severe acute respiratory syndrome coronavirus 2 (SARS‐CoV‐2) is a novel coronavirus that causes coronavirus disease 2019 (COVID‐19) [1]. COVID‐19 emerged in 2019 in Wuhan, China [2], and has since spread globally, causing a pandemic. The symptoms of COVID‐19 vary among individuals, ranging from mild respiratory symptoms to severe lung injury, multiorgan dysfunction, and death [3, 4, 5, 6]. Increasing evidence suggests that disease severity depends not solely on viral infection, but also on an excessive host proinflammatory response, whereby high concentrations of proinflammatory cytokines result in an unfavorable immune response and induce tissue damage [7, 8]. The events leading to excessive proinflammatory responses are not completely understood. Therefore, it is necessary to elucidate the mechanisms that are triggered by SARS‐CoV‐2 to induce innate and adaptive immune responses.

Innate immune cells express PRRs that recognize PAMPs and subsequently orchestrate an immune response against pathogens [9]. DCs are essential immune cells that function as a bridge between innate and adaptive immunity. DCs express various PRR families such as TLRs and cytosolic RIG‐I‐like receptors that are triggered upon virus interaction or infection [10]. DCs are therefore essential during SARS‐CoV‐2 infection to sense infection and instruct T and B cells for efficient antiviral immune responses. However, it is unclear whether and how SARS‐CoV‐2 is sensed by DCs.

SARS‐CoV‐2 Spike (S) protein uses angiotensin converting enzyme 2 (ACE2) [11, 12] as receptor for infection. However, besides interacting with ACE2, recent in silico analyses suggest that the Spike (S) protein could also potentially interact with members of the TLR family, in particular TLR4 [13, 14]. TLR4 is abundantly expressed on DCs [15, 16], and therefore, TLR4 signaling could be involved in induction of proinflammatory mediators. Other studies using cell lines and SARS‐CoV‐2 S protein support a potential interaction of TLR4 with the S protein [17, 18, 19]. However, it remains unclear whether infectious SARS‐CoV‐2 virus is sensed by TLR4 and whether this interaction induces DC activation and initiation of immunity.

Here, we have investigated how SARS‐CoV‐2 is sensed by human DCs. Neither recombinant S protein, SARS‐CoV‐2 pseudovirus, nor a primary SARS‐CoV‐2 isolate induced immunity in TLR4‐expressing cell lines or DCs, indicating that TLR4 or other extracellular TLRs are not involved in SARS‐CoV‐2 infection. However, ectopic expression of ACE2 on DCs led to infection by SARS‐CoV‐2 and induction of type I IFN and cytokines. These data imply that intracellular PRRs rather than transmembrane TLRs are involved in instigating an immune response against SARS‐CoV‐2.

## Results

### SARS‐CoV‐2 S protein does not trigger TLR4

**Figure 1:**
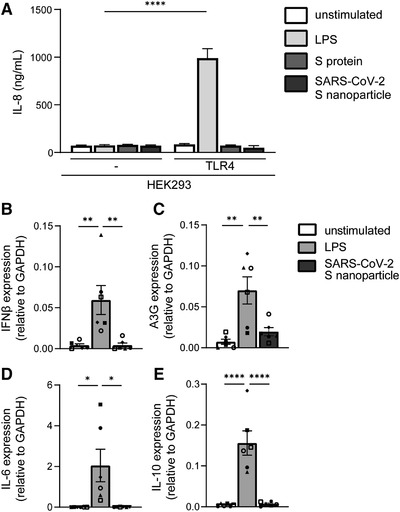
S protein and SARS‐CoV‐2 S nanoparticles do not trigger TLR4. (A) 293 cells or 293/TLR4 cells were exposed to LPS, SARS‐CoV‐2 S protein or S nanoparticles for 24 h. IL‐8 production was determined by ELISA. (B‐E) Primary dendritic cells were exposed to LPS or SARS‐CoV‐2 S nanoparticles for 8 h. Expression of IFN‐β (B), A3G (C), IL‐6 (D), and IL‐10 (E) was determined with qPCR. Data show the mean values and SEM. Statistical analysis was performed using (A) two‐way ANOVA with Šidák's multiple comparisons test or (B‐E) one‐way ANOVA with Tukey's multiple comparisons test. Data represent six replicates obtained in three separate experiments (A), or experiments performed with six donors in three independent experiments, with each symbol representing a different donor (B‐E). ****p < 0.0001, ***p < 0.001; **p < 0.01; *p < 0.05.

To assess whether TLR4 acts as a sensor of S protein of SARS‐CoV‐2, we treated a TLR4‐expressing HEK293 cell line (293/TLR4) with SARS‐CoV‐2 recombinant S protein or S nanoparticles [20] and determined activation by measuring IL‐8. Neither S protein nor S nanoparticles induced IL‐8 secretion by 293/TLR4 cells, in contrast to the positive control LPS (Fig. 1A). The parental 293 cells did not induce IL‐8 upon treatment with S protein or S nanoparticles and LPS. These data suggest that S protein of SARS‐CoV‐2 does not trigger TLR4.

Primary monocyte‐derived DCs express TLR4 but also other TLRs [21]. We therefore exposed primary human DCs to SARS‐CoV‐2 S nanoparticles and assessed cytokine production by qPCR. Treatment of DCs with S nanoparticles neither induces type I IFN nor cytokines (Fig. 1B‐E). The positive‐control LPS induced IFN‐β (Fig. 1B) and the IFN‐stimulated gene (ISG) APOBEC3G (A3G) (Fig. 1C) as well as cytokines IL‐6 and IL‐10 (Fig. 1D and E). These data strongly suggest that S protein from SARS‐CoV‐2 does not trigger extracellular TLRs on DCs.

### SARS‐CoV‐2 virus particles do not trigger TLR4

**Figure 2:**
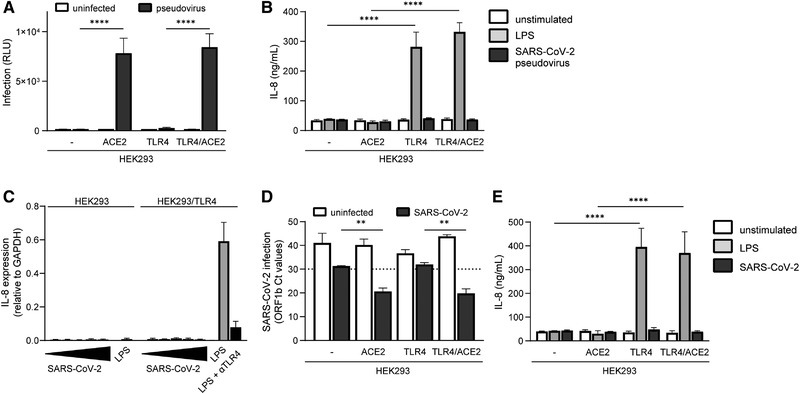
SARS‐CoV‐2 virus particles do not trigger TLR4. (A‐B) ACE2‐positive and ‐negative 293 and 293/TLR4 cells were exposed to SARS‐CoV‐2 pseudovirus and infection was determined after 3 days by measuring luciferase activity (A), and IL‐8 production was measured after 24 h by ELISA (B). (C) 293 and 293/TR4 cells were exposed to increasing titers of SARS‐CoV‐2, or LPS in the absence or presence of anti‐TLR4 antibodies, and IL‐8 production was determined after 24 h by qPCR. Increasing titers are indicated by a bar, ranging from TCID100 (narrow) to TCID100.000 (wide). (D‐E) ACE2‐positive and ‐negative 293 and 293/TLR4 cells were exposed to a primary SARS‐CoV‐2 isolate and infection was determined after 24 h by measuring the viral gene ORFb1 expression in supernatant by qPCR (D) and IL‐8 production was measured after 24 h by ELISA (E). Data show the mean values and SEM. Statistical analysis was performed using two‐way ANOVA with Šidák's (A) or Tukey's (B, D‐E) multiple comparisons test. Data represent nine replicates obtained in three separate experiments (A‐B), or three separate experiments (C‐E). ****p < 0.0001; **p < 0.01. RLU, relative light units.

To assess whether TLR4 plays a role in SARS‐CoV‐2 entry and replication, we ectopically expressed ACE2 on 293 and 293/TLR4 cell lines and infected the cells with SARS‐CoV‐2 pseudovirus that expresses the full‐length S glycoprotein from SARS‐CoV‐2 and contains a luciferase reporter gene [22]. Infection was determined by measuring luciferase activity. SARS‐CoV‐2 pseudovirus infected ACE2‐positive 293 and 293/TLR4 cells but not the parental 293 and 293/TLR4 cells (Fig. 2A). TLR4 expression did not affect infection, as infection was comparable between 293/ACE2 and 293/TLR4/ACE2 cells.

Next, we investigated whether SARS‐CoV‐2 pseudovirus activates TLR4. SARS‐CoV‐2 pseudovirus neither induces IL‐8 in parental 293 nor in 293/TLR4 cells (Fig. 2B). Moreover, ACE2 expression did not induce activation as exposure of ACE2‐positive 293 and 293/TLR4 cells to SARS‐CoV‐2 pseudovirus did not lead to IL‐8 production (Fig. 2B). These data further support the findings that S protein from SARS‐CoV‐2 does not trigger TLR4 and also show that ACE2 does not affect TLR4 signaling.

Next, we performed a serial dilution with a primary SARS‐CoV‐2 isolate (hCoV‐19/Italy) on 293 and 293/TLR4 cells to determine whether high virus concentrations are able to induce TLR4. Neither 293 nor 293/TLR4 cells expressed IL‐8 upon exposure to the primary SARS‐CoV‐2 isolate, suggesting that high virus concentrations do not trigger TLR4 (Fig. 2C). Next, we treated either ACE2‐positive or ‐negative 293 and 293/TLR4 cells with the primary SARS‐CoV‐2 isolate and determined infection and activation. Infection was determined by measuring virus particles in the supernatant by qPCR. As expected, both 293/ACE2 and 293/TLR4/ACE2 cells were productively infected at similar levels by SARS‐CoV‐2, in contrast to ACE2‐negative 293 and 293/TLR4 cells (cut‐off *Ct* values > 30), (Fig. 2D). Neither ACE2‐positive nor ‐negative 293 and 293/TLR4 cells expressed any IL‐8 upon exposure to the primary SARS‐CoV‐2 isolate (Fig. 2E). These data strongly suggest that TLR4 does not sense infectious SARS‐CoV‐2 virus particles.

### Infectious SARS‐CoV‐2 does not activate DCs

**Figure 3:**
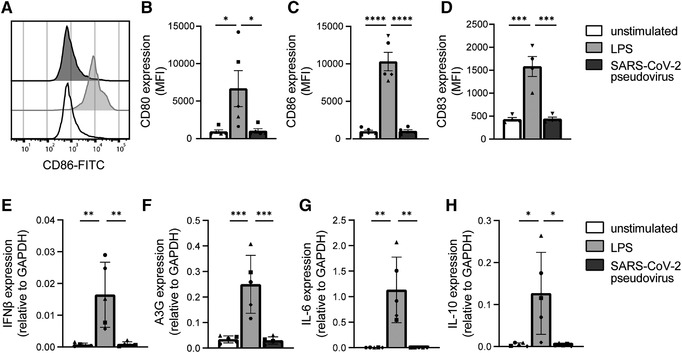
SARS‐CoV‐2 pseudovirus does not activate dendritic cells. (A‐D) Primary DCs were exposed to LPS or SARS‐CoV‐2 pseudovirus and maturation and cytokine production was determined after 24 and 6 h, respectively. (A) Representative histogram of CD86 expression. (B‐D) Cumulative flow cytometry data of CD80 (B), CD86 (C), and CD83 (D) expression. (E‐H) mRNA levels of IFN‐β (E), A3G (F), IL‐6 (G), and IL‐10 (H) were determined with qPCR. Data show the mean values and SEM. Statistical analysis was performed using one‐way ANOVA with Tukey's multiple comparisons test. Data represent five donors analyzed in three separate experiments (B‐C, E‐H), or four donors analyzed in two separate experiments (D), with each symbol representing a different donor. ****p < 0.0001; ***p < 0.001; **p < 0.01; *p < 0.05. MFI, mean fluorescence intensity.

Subsequently, we examined whether SARS‐CoV‐2 pseudovirus induces DC maturation and cytokine production. DCs do not express ACE2 and we have previously shown that SARS‐CoV‐2 pseudovirus does not infect DCs [23, 24]. We investigated the maturation and cytokine production by DCs stimulated with SARS‐CoV‐2 pseudovirus. Exposure of DCs to SARS‐CoV‐2 pseudovirus neither induces expression of costimulatory markers CD80 and CD86 nor maturation marker CD83, in contrast to LPS (Fig. 3A‐D, Supporting information Fig. S1). Moreover, SARS‐CoV‐2 pseudovirus did not induce any cytokines, in contrast to LPS (Fig. 3E‐H). These data indicate that the S protein expressed by SARS‐CoV‐2 pseudovirus does not activate DCs.

**Figure 4:**
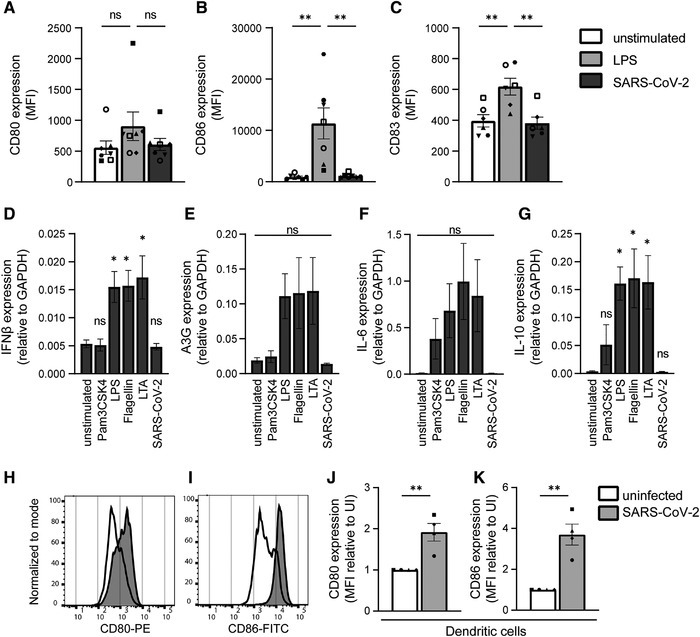
Primary SARS‐CoV‐2 isolate does not activate DCs. (A‐C) Primary DCs were exposed to LPS or primary SARS‐CoV‐2 isolate and DC maturation was measured after 24 h by flow cytometry. Cumulative flow cytometry data of CD80 (A), CD86 (B), and CD83 (C) expression. (D‐G) Primary DCs were exposed to different TLR agonists or primary SARS‐CoV‐2 isolate and mRNA levels of IFN‐β (D), A3G (E), IL‐6 (F), and IL‐10 (G) were determined with qPCR. (D‐G) Data are compared to the unstimulated condition. (H‐K) Primary DCs were cocultured with VeroE6 cells infected by SARS‐CoV‐2 and DC maturation was determined after 24 h by measuring expression of CD80 and CD86. (H‐I) Representative histograms of CD80 (H) and CD86 (I) expression. (J‐K) Cumulative flow cytometry data of CD80 (J) and CD86 (K) expression. Data is relative to the uninfected condition (UI). Data show the mean values and SEM. Statistical analysis was performed using one‐way ANOVA with Tukey's multiple comparisons test (A‐G), or using an unpaired student's *t*‐test (J‐K). Data represent seven donors (A‐B) or six donors (C) analyzed in four experiments; or five donors analyzed in three separate experiments (D‐G); or four donors analyzed in two separate experiments (J‐K), with each symbol representing a different donor. **p < 0.01; *p < 0.05; MFI, mean fluorescence intensity; ns, nonsignificant; UI, uninfected.

Next, we exposed DCs to a primary SARS‐CoV‐2 isolate and determined DC maturation and cytokine production. We have previously shown that DCs do not become infected by primary SARS‐CoV‐2 [23]. Exposure of DCs to the primary SARS‐CoV‐2 isolate neither induces expression of CD80, CD86, nor of CD83, whereas LPS induced expression of CD83 and CD86 (Fig. 4A‐C).

Next, we investigated cytokine induction by DCs after exposure to primary SARS‐CoV‐2 isolate or agonists for extracellular TLRs (TLR1/2, TLR2/6, TLR4, and TLR5). LPS, flagellin, and lipoteichoic acid induced type I IFN responses as well as cytokines, whereas Pam3CSK4 only induced cytokines (Fig. 4D‐G). However, exposure of DCs to the primary SARS‐CoV‐2 isolate did not lead to induction of type I IFN responses or cytokines (Fig. 4D‐G). Therefore, these data strongly indicate that primary SARS‐CoV‐2 virus particles are not sensed by any extracellular PRRs on DCs such as TLR2, TLR4, and TLR5.

Although SARS‐CoV‐2 did not directly activate DCs, we investigated whether DCs become activated indirectly by SARS‐CoV‐2‐infected cells. Therefore, DCs were cocultured with SARS‐CoV‐2 infected VeroE6 cells and DC activation was determined. Strikingly, coculture of DCs with SARS‐CoV‐2‐infected, but not uninfected VeroE6 cells induced expression of costimulatory molecules CD80 and CD86 (Fig. 4H‐K). These data support a role for indirect activation of DCs by infected cells during SARS‐CoV‐2 infection.

### Ectopic ACE2 expression on DCs results in SARS‐CoV‐2 infection and immune activation

**Figure 5:**
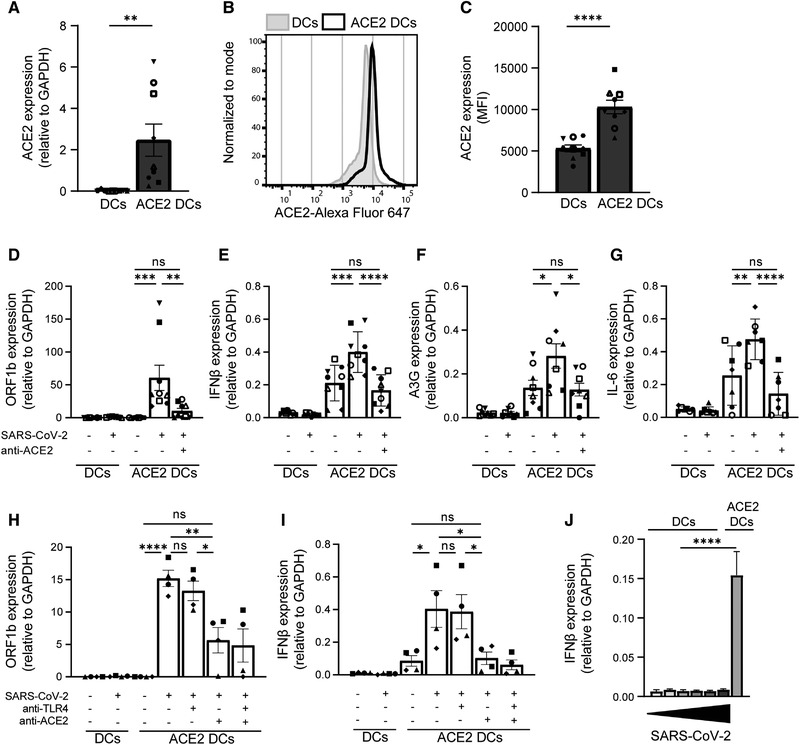
Ectopic expression of ACE2 on DCs results in infection and induction of immune responses. (A‐C) Ectopic expression of ACE2 on primary DCs was determined by qPCR and flow cytometry. (A) Cumulative qPCR data of ACE2 expression on DCs. (B) Representative histogram of ACE2 expression on DCs. (C) Cumulative flow cytometry data of ACE2 expression. (D‐G) ACE2‐positive and ‐negative DCs were exposed to primary SARS‐CoV‐2 isolate in presence or absence of blocking antibodies against ACE2. Infection (D) and mRNA levels of IFN‐β (E), A3G (F), and IL‐6 (G) were determined with qPCR. (H‐I) ACE2‐positive and ‐negative DCs were exposed to primary SARS‐CoV‐2 isolate in presence of blocking antibodies against TLR4 and ACE2. Infection (H) and mRNA levels of IFN‐β (I) were determined with qPCR. (J) ACE2‐ negative DCs were exposed to increasing titers of primary SARS‐CoV‐2 isolate for 24 h and compared to ACE2‐positive DCs infected with TCID1000, and mRNA levels of IFN‐β were determined by qPCR. Increasing titers are indicated by a bar, ranging from TCID100 (narrow) to TCID100.000 (wide). Data show the mean values and SEM. Statistical analysis was performed using (A, C) unpaired student's *t*‐test or (D‐I) one‐way ANOVA with Tukey's multiple comparisons test. Data represent nine donors (A, C‐F) or seven donors (G) obtained in five separate experiments, or four donors (H‐J) obtained in two separate experiments, with each symbol representing a different donor. ****p < 0.0001; ***p < 0.001; **p < 0.01; *p < 0.05; MFI, mean fluorescence intensity; ns, nonsignificant.

Next, we investigated whether infection of DCs after ectopic expression of ACE2 with primary SARS‐CoV‐2 isolate would induce immune responses. DCs do not express ACE2, but transfection with ACE2 plasmid resulted in ACE2 mRNA and surface expression (Fig. 5A‐C). Next, both DCs and ACE2‐expressing DCs were exposed to the primary SARS‐CoV‐2 isolate for 24 h in presence or absence of blocking antibodies against ACE2. ACE2‐expressing DCs were infected by SARS‐CoV‐2 and infection was blocked by antibodies against ACE2 (Fig. 5D). Notably, infection of DCs with SARS‐CoV‐2 induced transcription of IFN‐β (Fig. 5E) as well as the IFN‐stimulated gene A3G (Fig. 5F). Infection also induced proinflammatory cytokine IL‐6 (Fig. 5G). Both type I IFN responses and IL‐6 were abrogated by blocking infection using ACE2 antibodies. Although the transfection procedure itself slightly activates DCs, SARS‐CoV‐2 infection significantly increased DC activation, which was abrogated by blocking ACE2. These data strongly suggest that DC activation of ACE2‐expressing DCs is due to SARS‐CoV‐2 infection.

It has been described that TLR4 not only induces signaling pathways from the plasma membrane, but can also be internalized to the endosomal pathway to induce alternative signaling [25]. To investigate whether ACE2‐mediated internalization of SARS‐CoV‐2 triggers endosomal TLR4, we blocked TLR4 upon infection. Both DCs and ACE2‐expressing DCs were exposed to the primary SARS‐CoV‐2 isolate in presence or absence of blocking antibodies against TLR4 and ACE2. ACE2‐expressing DCs were infected by SARS‐CoV‐2 and both infection and IFN‐β production was blocked by antibodies against ACE2, but not by antibodies against TLR4 (Fig. 5H and I), suggesting that endosomal TLR4 triggering is not involved in the observed SARS‐CoV‐2‐induced immune activation. Moreover, higher concentrations of the primary SARS‐CoV‐2 isolate did not induce type I IFN responses in DCs compared to ACE2‐expressing DCs (Fig. 5J). Taken together, these data strongly indicate that infection is required to induce cytokine responses by DCs and suggest that intracellular PRRs rather than extracellular TLRs are involved in sensing SARS‐CoV‐2 and instigating immune responses against SARS‐CoV‐2.

## Discussion

SARS‐CoV‐2 has established itself as a contagious human respiratory pathogen, which can trigger a robust inflammatory cytokine response [8]. However, it remains largely unknown whether innate immune receptors are involved in the onset of immune responses against SARS‐CoV‐2. TLR4 has been suggested to play a role in sensing SARS‐CoV‐2 and inducing a strong immune response [13, 14]. Here, our data suggest that SARS‐CoV‐2 by itself is not recognized by TLR4, as neither a TLR4‐expressing 293 cell line nor primary DCs were activated by exposure to recombinant S protein, SARS‐CoV‐2 pseudovirus or primary SARS‐CoV‐2 virus particles. Ectopic expression of ACE2 on primary DCs allowed infection with primary SARS‐CoV‐2. Notably, productive infection of ACE2‐positive DCs induced type I IFN and cytokine responses, which were abrogated by blocking ACE2. Our data therefore suggest that SARS‐CoV‐2 virus particles are not sensed by extracellular TLRs, including TLR4, but that infection via ACE2 is required.

Other studies have reported that SARS‐CoV‐2 S protein triggers TLR4, and TLR2 and TLR6 are suggested to interact with the S protein [13, 14, 17–19, 26]. However, neither a TLR4‐expressing 293 cell line nor primary DCs were activated by recombinant S proteins. It is possible that contamination during the purification process of recombinant proteins might induce activation and explain the differences. Therefore, we have also investigated immune activation by SARS‐CoV‐2 pseudovirus and infectious primary SARS‐CoV‐2 isolates. However, neither TLR4‐expressing 293 cells nor primary DCs were activated by pseudovirus or a primary isolate of SARS‐CoV‐2, even at high virus concentrations. Therefore, our data strongly suggest that S protein expressed by SARS‐CoV‐2 does not trigger TLR4. Differences between our findings and those published might be due to different S protein preparations, purity of recombinant proteins, or cell models. Most studies have used cell lines whereas we have used primary monocyte‐derived DCs, which express high levels of TLR4, and are sensitive to TLR4 agonists. Monocyte‐derived DCs are present in human lungs [27, 28] and monocytes infiltrating the lungs can differentiate into monocyte‐derived DCs after pathogen exposure [29, 30], which further supports the relevance of monocyte‐derived DCs to study TLR4 function in SARS‐CoV‐2 infection.

Monocyte‐derived DCs do not express ACE2 [24] and did not become infected by SARS‐CoV‐2, suggesting that the inability of primary SARS‐CoV‐2 to activate DCs strongly implies that SARS‐CoV‐2 is not sensed by TLR4 or other extracellular PRRs. Notably, ectopically expressing ACE2 on DCs led to infection and the production of cytokines, indicating that replication of SARS‐CoV‐2 triggers cytosolic sensors. Indeed, studies suggest that intracellular viral sensors, such as RIG‐I or MDA5, are involved in SARS‐CoV‐2 infection [31, 32, 33]. Our data, therefore, support an important role for infection by SARS‐CoV‐2 in inducing immune activation and imply that infection of immune cells, such as APCs, is essential to induce immunity. Therefore, it is important to identify ACE2‐positive DC subsets and macrophages, since these APCs could be sensitive to infection and thereby orchestrate adaptive immunity. However, in the absence of DC infection, epithelial cell infection and subsequent inflammation and tissue damage might account for initial immune activation as release of PAMPs and DAMPs by these infected cells might activate ACE2‐negative DCs [34]. Notably, coculture of DCs with SARS‐CoV‐2‐infected cells led to activation of DCs, supporting a role for indirect activation of DCs by infected cells. It remains unclear whether these secondary signals are able to correctly instruct DCs and this might underlie the strong inflammatory responses observed during COVID‐19. Our finding that SARS‐CoV‐2 is not recognized by TLR4 might therefore be an escape mechanism leading to inefficient DC activation and subsequent aberrant inflammatory responses.

It has been suggested that worsening of disease in COVID‐19 patients coincides with the activation of the adaptive immune response, 1–2 weeks after infection [8]. Since DCs have a bridging function to activate the adaptive immune response, it is important to study DCs in the context of COVID‐19. Our research suggests that ACE2‐negative DCs are not properly activated by infectious SARS‐CoV‐2. Moreover, our data suggest that SARS‐CoV‐2 is able to escape from extracellular TLRs that are one of the most important PRR families crucial for induction of innate and adaptive immunity, and further research will show whether the lack of TLR activation underlies observed inflammation during COVID‐19.

## Materials and methods

### Cell lines

The Simian kidney cell line VeroE6 (ATCC CRL‐1586) was maintained in CO_2_ independent medium (Gibco Life Technologies, Gaithersburg, Md.) supplemented with 10% FCS, 2 mM l‐glutamine, and 1% penicillin/streptomycin. Cultures were maintained at 37°C without CO_2_.

Human embryonic kidney cells (HEK293) were maintained in IMDM (Gibco) supplemented with 10% FCS and 1% penicillin/streptomycin (Invitrogen). HEK293 cells stably transfected with TLR4 cDNA (HEK/TLR4) were a kind gift from D. T. Golenbock [15]. HEK293 and HEK/TLR4 cells were transiently transfected with pcDNA3.1(‐)hACE2 (Addgene plasmid #1786) to generate HEK/ACE2 or HEK/TLR4/ACE2 cell lines. Transfection was performed using lipofectamine LTX and PLUS reagent (Invitrogen) according to the manufacturer's protocol. After 24 h, cells were split and seeded into flat‐bottom 96‐well plates (Corning) and left to attach for 24 h, before performing further experiments. Cultures were maintained at 37°C and 5% CO_2_. Before infection with the SARS‐CoV‐2 isolate (described below), media was exchanged for CO_2_‐independent media, since infection with a SARS‐CoV‐2 primary isolate occurs under CO_2_‐negative conditions. Human ACE2‐expressing cell lines were analyzed for ACE2 expression via quantitative real‐time PCR.

### Primary cells

This study was performed in accordance with the ethical principles set out in the Declaration of Helsinki and was approved by the institutional review board of the Amsterdam University Medical Centers, location AMC Medical Ethics Committee and the Ethics Advisory Body of Sanquin Blood Supply Foundation (Amsterdam, Netherlands). Human CD14^+^ monocytes were isolated from the blood of healthy volunteer donors (Sanquin blood bank) and subsequently differentiated into monocyte‐derived DCs. The isolation from buffy coats was done by density gradient centrifugation on Lymphoprep (Nycomed) and Percoll (Pharmacia). After separation by Percoll, the isolated monocytes were cultured in RPMI 1640 (Gibco) supplemented with 10% FCS, 2mM L‐glutamin (Invitrogen), and 10 U/mL penicillin and 100 μg/mL streptomycin, containing the cytokines IL‐4 (500 U/mL) and GM‐CSF (800 U/mL) (both Gibco) for differentiation into DCs. After 4 days of differentiation, DCs were seeded at 1 × 10^6^/mL in a 96‐well plate (Greiner), and after 2 days of recovery, DCs were stimulated or infected as described below.

Alternatively, monocyte‐derived DCs that were transfected with hACE2 were seeded at 0.5 × 10^6^ cells/mL in a six‐well plate and transfection was performed with lipofectamine LTX and PLUS reagents (Invitrogen) according to the manufacturer's instructions for primary cells. After 24 h, cells were seeded at 1 × 10^6^/mL in a 96‐well plate and after 24 h of recovery, they were infected with primary SARS‐CoV‐2 isolate.

### SARS‐CoV‐2 pseudovirus production

For production of single‐round infection viruses, human embryonic kidney 293T/17 cells (ATCC, CRL‐11268) were cotransfected with an adjusted HIV‐1 backbone plasmid (pNL4‐3.Luc.R‐S‐) containing previously described stabilizing mutations in the capsid protein (PMID: 12547912) and firefly luciferase in the *nef* open reading frame (1.35 μg) and pSARS‐CoV‐2 expressing SARS‐CoV‐2 S protein (0.6 μg) (GenBank; MN908947.3) [22]. Transfection was performed in 293T/17 cells using genejuice (Novagen, USA) transfection kit according to manufacturer's protocol. At day 3 or 4, pseudotyped SARS‐CoV‐2 virus particles were harvested and filtered over a 0.45 μm nitrocellulose membrane (SartoriusStedim, Gottingen, Germany). SARS‐CoV‐2 pseudovirus productions were quantified by p24 ELISA (Perkin Elmer Life Sciences).

### SARS‐CoV‐2 (primary isolate) virus production

The following reagent was obtained from Dr. Maria R. Capobianchi through BEI Resources, NIAID, NIH:SARS‐related coronavirus 2, Isolate Italy‐INMI1, NR‐52284, originally isolated in January 2020 in Rome, Italy. VeroE6 cells (ATCC CRL‐1586) were inoculated with the SARS‐CoV‐2 isolate and used for reproduction of virus stocks. Cytopathic effect formation was closely monitored and virus supernatant was harvested after 48 h. Tissue culture infectious dose (TCID50) was determined on VeroE6 cells by MTT assay 48 h after infection. Loss of MTT staining as determined by spectrometer is indicative of cell death. The virus titer was determined as TCID50/mL and calculated based on the Reed Muench method [35], as described before [23].

### Stimulation and infection

HEK293 and transfected derivatives were left unstimulated or stimulated for 24 h with 10 ng/mL LPS from *Salmonella* (Sigma), 30 μg/mL isolated S protein, 10 μg/mL S nanoparticle, or with pseudotyped or authentic SARS‐CoV‐2, as specified below. DCs were left unstimulated, or stimulated with 10 μg/mL Pam3CSK4 (Invivogen), 10 ng/mL LPS from *Salmonella typhosa* (Sigma), 10 μg/mL flagellin from *Salmonella typhimurium* (Invivogen), 10 μg/mL LTA from *Staphylococcus aureus* (Invivogen), pseudotyped virus, or SARS‐CoV‐2. Blocking of ACE2 or TLR4 was performed with 8 μg/mL anti‐ACE2 (R&D systems) or 10 μg/mL anti‐TLR4 (clone 7E3, Hycult) for 30 min at 37°C before adding stimuli. Monocyte‐derived DCs do not express ACE2 and are, therefore, not infected. Therefore, pseudovirus stimulation was performed for 6 h, after which, the cells were lysed for mRNA analysis of cytokine production. DCs ectopically expressing ACE2 were stimulated for 24 h with virus, before the cells were lysed for mRNA analysis of cytokine production. Also, cells were stimulated for 24 h and fixed for 30 min with 4% paraformaldehyde, after which the expression of maturation markers was assessed with flow cytometry.

For the pseudovirus infection assays, HEK293 or 293/TLR4 cell lines and DCs were exposed at 95 ng/mL and 191.05 ng/mL of SARS‐CoV‐2 pseudovirus, respectively. Viral protein production was quantified after 3 days at 37°C by measuring luciferase reporter activity. Luciferase activity was measured using the luciferase assay system (Promega, USA) according to the manufacturer's instructions.

For the primary SARS‐CoV‐2 infection assays, HEK293 or HEK/TLR4 cell lines and DCs were exposed to the SARS‐CoV‐2 isolate (hCoV‐19/Italy) at different TCIDs (100 and 1000; MOI 0.0028‐0.028) for 24 h at 37°C. After 24 h, cell supernatant was taken and DCs were lysed for isolation of viral RNA. Also, the HEK293/ACE2 and HEK/TLR4/ACE2 cell lines were exposed to the SARS‐CoV‐2 isolate (hCoV‐19/Italy) at TCID 100 (MOI 0.0028) for 24 h at 37°C. After 24 h, the cells were washed three times and new media was added. After 48 h, cell supernatant was harvested and the cells were lysed to investigate productive infection.

### RNA isolation and quantitative real‐time PCR

Cells exposed to SARS‐CoV‐2 pseudovirus were lysed and mRNA was isolated with the mRNA Catcher PLUS Purification Kit (ThermoFisher). Subsequently, cDNA was synthesized with a reverse‐transcriptase kit (Promega). RNA of cells exposed to SARS‐CoV‐2 WT was isolated with the QIAamp Viral RNA Mini Kit (Qiagen) according to the manufacturer's protocol. cDNA was synthesized with the M‐MLV reverse‐transcriptase kit (Promega) and diluted one in five before further application. PCR amplification was performed in the presence of SYBR green (ThermoFisher) in a 7500 Fast Realtime PCR System (ABI). Specific primers were designed with Primer Express 2.0 (Applied Biosystems). The ORF1b primers used were as described before [36]. The normalized amount of target mRNA was calculated from the *Ct* values obtained for both target and household mRNA with the equation *Nt* = 2*^Ct^* ^(GAPDH)‐Ct(target)^. The following primers were used:

GAPDH: F_CCATGTTCGTCATGGGTGTG; R_GGTGCTAAGCAGTTGGTGGTG; TLR4: F_CTGCAATGGATCAAGGACCAG; R_CCATTCGTTCAACTTCCACCA; ACE2: F_GGACCCAGGAAATGTTCAGA; R_ GGCTGCAGAAAGTGACATGA; ORF1b: F_TGGGGTTTTACAGGTAACCT; R_AACACGCTTAACAAAGCACTC; IL‐8: F_TGAGAGTGGACCACACTGCG; R_TCTCCACAACCCTCTGCACC; IFNB: F_ACAGACTTACAGGTTACCTCCGAAAC; R_CATCTGCTGGTTGAAGAATGCTT; APOBEC3G: F_TTGAGCCTTGGAATAATCTGCC; R_TCGAGTGTCTGAGAATCTCCCC; IL‐6: F_TGCAATAACCACCCCTGACC; R_TGCGCAGAATGAGATGAGTTG; IL‐10: F_GAGGCTACGGCGCTGTCAT; R_CCACGGCCTTGCTCTTGTT

### ELISA

Cell supernatants were harvested after 24 h of stimulation and secretion of IL‐8 was measured by ELISA (eBiosciences) according to the manufacturer's instructions. OD450 nm values were measured using a BioTek Synergy HT. Supernatant containing SARS‐CoV‐2 pseudovirus was inactivated with 0.1% triton and supernatant containing SARS‐CoV‐2 was inactivated with 1% triton before performing ELISA.

### Flow cytometry

For cell surface staining, cells were incubated in 0.5% PBS‐BSA (Sigma‐Aldrich) containing antibodies for 30 min at 4°C. Single‐cell measurements were performed on a FACS Canto flow cytometer (BD Biosciences) and FlowJo V10 software (TreeStar) was used to analyze the data. The antibody clones used are: CD86 (2331 (FUN‐1), BD Pharmingen), CD80 (L307.4, BD Pharmingen), CD83 (HB15e, BD Pharmingen), ACE2 (AF933, R&D Systems), goat‐IgG (AB‐2535864, ThermoFisher Scientific), donkey‐anti‐goat (A‐21447, ThermoFisher Scientific). For each experiment, live cells were gated on FSC and SSC and analyzed further with the markers mentioned (Supporting information Fig. S1). The authors adhered to the guidelines for the use of flow cytometry and cell sorting in immunological studies [37].

### Statistics

Graphpad Prism version8 (GraphPad Software) was used to generate all graphs and for statistical analyses. Statistics were performed using a Student's *t* test for pairwise comparisons. Multiple comparisons within groups were performed using an RM one‐way ANOVA with a Tukey's multiple comparisons test, or two‐way ANOVA with a Tukey's or Šidák's multiple comparisons test, where indicated. *p* < 0.05 were considered statistically significant.

## Conflict of Interests

All authors declare no commercial or financial conflict of interest.

## Ethics approval statement

This study was performed in accordance with the ethical principles set out in the Declaration of Helsinki and was approved by the institutional review board of the Amsterdam University Medical Centers, location AMC Medical Ethics Committee and the Ethics Advisory Body of Sanquin Blood Supply Foundation (Amsterdam, Netherlands).

## Author contributions

LEHvdD and MBJ designed experiments; LEHvdD, MBJ, JE, and JLvH performed the experiments; PJMB, MB, ACvN, NAK, MJvG, and RWS contributed essential research materials and scientific input. LEHvdD, MBJ, and TBHG analyzed and interpreted data; LEHvdD, MBJ, and TBHG wrote the manuscript with input from all listed authors. TBHG supervised all aspects of this study.

### Peer review

The peer review history for this article is available at https://publons.com/publon/10.1002/eji.202149656.

**Abbreviations**
ACE2: angiotensin converting enzyme 2
COVID‐19: coronavirus disease 19
HEK293: Human embryonic kidney cells
S: Spike ()
SARS‐CoV‐2: severe acute respiratory syndrome coronavirus 2

## Supporting information

Supporting information

## Data Availability

The data generated during this study are available from the corresponding author on reasonable request.
