# Supplementary material for: SARS‐CoV‐2 infection activates dendritic cells via cytosolic receptors rather than extracellular TLRs
Source: Eur J Immunol. 2022 Feb 16;52(4):646–55. doi: 10.1002/eji.202149656 (PMC9015339; doi:10.1002/eji.202149656)
Supplement: Supplementary file 1 — Supporting information [file EJI-52--s001.pdf]

Supplemental information

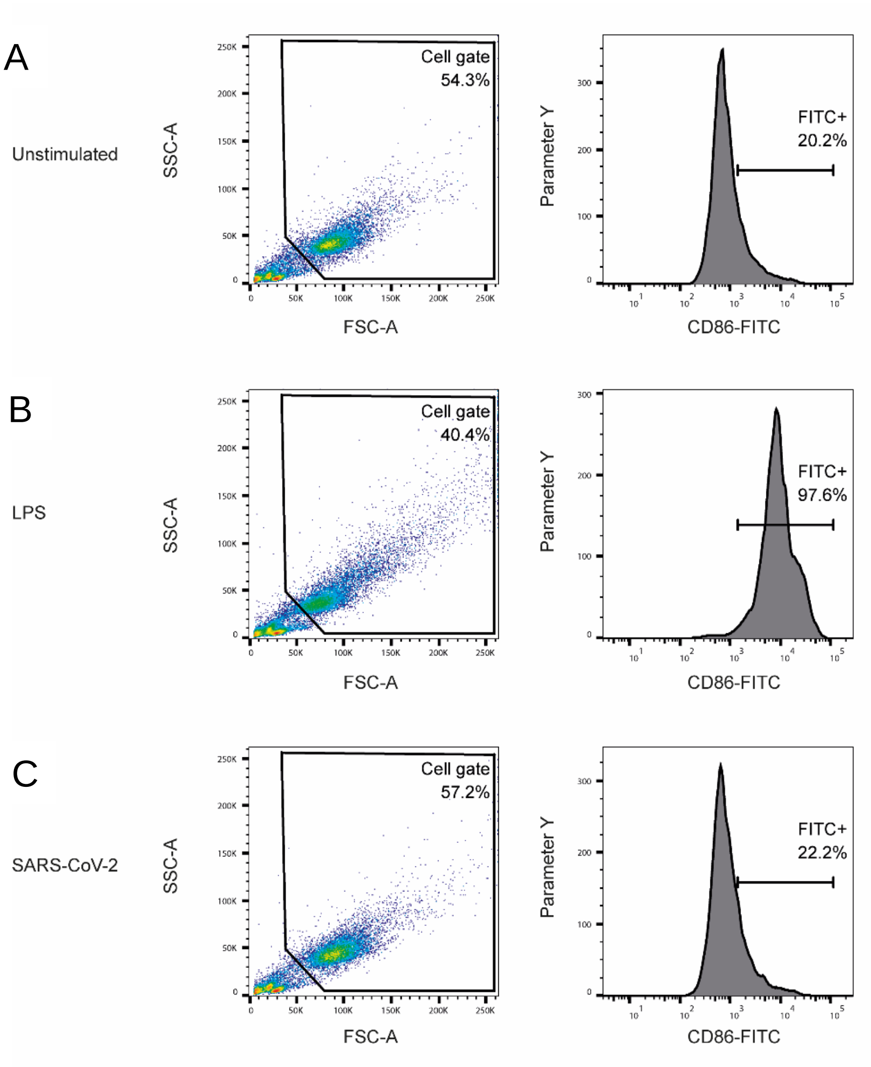

Supplemental Figure 1: Dendritic cell gating strategy

After exposure to different stimuli, dendritic cells were harvested, fixed, and stained with antibodies against various markers and analyzed by flow cytometry. (A-C) Representative flow cytometry plots of one donor stimulated with medium (A), LPS (B), or SARS-CoV-2 (C). The percentage of selected cells is depicted in the upper right corner of the dot plot, and the expression of CD86 was plotted in a histogram. Histograms show the percentages of CD86-FITC-positive cells.
